# Endothelial Mitochondrial Dysfunction in INOCA and Coronary Microvascular Dysfunction: Mechanisms, Sex Differences, and Therapeutic Implications

**DOI:** 10.3390/jcdd13070321

**Published:** 2026-07-10

**Authors:** Roko Santic, Lovre Martinovic, Marko Kumric, Nikola Pavlovic, Dinko Martinovic, Lovre Jukic, Zenon Pogorelic, Josko Bozic

**Affiliations:** 1 Department of Pathophysiology, University of Split School of Medicine, 21000 Split, Croatia; roko.santic@mefst.hr (R.S.); lovre.martinovic@mefst.hr (L.M.); marko.kumric@mefst.hr (M.K.); nikola.pavlovic@mefst.hr (N.P.); lj72091@mefst.hr (L.J.); josko.bozic@mefst.hr (J.B.); 2 Laboratory for Cardiometabolic Research, University of Split School of Medicine, Soltanska 2A, 21000 Split, Croatia; 3 Department of Maxillofacial Surgery, University Hospital of Split, Spinciceva 1, 21000 Split, Croatia; dmartinovic@kbsplit.hr; 4 Department of Maxillofacial Surgery, University of Split School of Medicine, Soltanska 2A, 21000 Split, Croatia; 5 Department of Pediatric Surgery, University Hospital of Split, Spinciceva 1, 21000 Split, Croatia; 6 Department of Surgery, University of Split School of Medicine, 21000 Split, Croatia

**Keywords:** INOCA, coronary microvascular dysfunction, endothelial mitochondrial dysfunction, endothelial dysfunction, coronary function testing, sex differences, nitric oxide, mitophagy, mitochondrial reactive oxygen species, mitochondrial biomarkers

## Abstract

Ischemia with non-obstructive coronary arteries (INOCA) and coronary microvascular dysfunction (CMD) are increasingly recognized causes of angina, reduced quality of life, and elevated cardiovascular risk, yet mechanistic heterogeneity complicates diagnosis and treatment. This narrative review synthesizes evidence from clinical guidelines, consensus documents, landmark trials, cohorts, mechanistic studies, and high-quality reviews identified through structured, non-exhaustive searches of PubMed/MEDLINE, Google Scholar, and major cardiovascular society documents. Current evidence indicates that endothelial mitochondria function primarily as signaling organelles, regulating reactive oxygen species, nitric oxide bioavailability, endothelium-dependent hyperpolarization, calcium signaling, inflammatory activation, mitophagy, and endothelial survival. Cardiometabolic risk factors, aging, chronic kidney disease, and postmenopausal hormonal changes may converge on mitochondrial quality-control and redox pathways, contributing to CMD susceptibility and sex-specific vulnerability. However, direct human evidence linking endothelial mitochondrial dysfunction causally to CMD defined by invasive coronary function testing remains limited. Coronary physiological testing and acetylcholine provocation are validated tools for CMD endotyping, whereas mitochondrial biomarkers remain investigational. Endotype-guided diagnosis and management remain central, while mitochondria-targeted strategies require prospective CMD-specific validation.

## 1. Introduction

Coronary artery disease (CAD) has long been conceptualized as a disease of epicardial obstruction, yet the majority of patients referred for elective coronary angiography are found to have no obstructive CAD, defined as absence of luminal stenosis ≥ 50% [1,2]. A substantial proportion of these individuals experience recurrent chest pain, objective evidence of myocardial ischemia, or reduced quality of life, constituting the clinical syndrome now broadly termed ischemia with non-obstructive coronary arteries (INOCA). When ischemia is detected by non-invasive testing but coronary angiography is normal, the term INOCA applies; when no obstructive disease is identified at angiography irrespective of ischemia documentation, the broader term angina with non-obstructive coronary arteries (ANOCA) is sometimes preferred [3]. These distinctions matter because they define patient populations in research and increasingly guide management pathways.

INOCA is not a rare or benign condition. Epidemiological data suggest that between 3 and 4 million people in the United States alone may be affected, and the syndrome accounts for a disproportionate share of repeated hospitalizations, invasive investigations, and impaired quality of life. Meta-analytic data indicate that patients with microvascular angina experience major adverse cardiovascular events at a rate of approximately 2.5 per 100 patient-years, while registry data confirm that objective CMD carries a risk of incident cardiovascular events at least fourfold higher than patients without coronary microvascular abnormality [4,5,6,7]. INOCA is more frequently recognized in women than in men, and women with INOCA report persistently impaired quality of life, with symptomatic burden comparable to patients with obstructive CAD [5,8]. Nevertheless, INOCA affects both sexes, and attributing the condition exclusively to women introduces a risk of diagnostic and therapeutic bias that the current evidence does not justify [3,9].

The 2024 European Society of Cardiology (ESC) Guidelines for the Management of Chronic Coronary Syndromes represent an important conceptual shift, explicitly recognizing that chronic coronary syndrome encompasses not only stable obstructive CAD but also nonobstructive disease including vasomotor disorders and CMD [1]. This evolution reflects a growing understanding that coronary pathophysiology extends beyond luminal stenosis to encompass endothelial function, smooth muscle reactivity, microvascular structure, and the interaction of these components with systemic cardiometabolic risk factors. The 2021 American Heart Association/American College of Cardiology Chest Pain Guideline similarly emphasizes the importance of evaluating vasomotor and microvascular mechanisms in patients presenting with chest pain without obstructive CAD [10].

Coronary microvascular dysfunction (CMD), as one of the principal mechanistic endotypes within INOCA, encompasses several distinct physiological abnormalities: reduced coronary flow reserve (CFR), elevated microvascular resistance, microvascular spasm, endothelial dysfunction, and mixed vasomotor dysfunction [3]. These endotypes may coexist and may overlap with diffuse nonobstructive atherosclerosis, vasospastic angina, hypertensive heart disease, diabetes, obesity, chronic kidney disease, and other systemic conditions. Identifying the dominant endotype is not merely academic; it is the foundation of endotype-guided treatment, as demonstrated by the CorMicA trial and the CorCMR study [11,12,13].

Clinically, the practical implication is that CMD endotypes should be interpreted and treated differently: reduced CFR and/or elevated microvascular resistance primarily direct attention to impaired vasodilator reserve, structural remodeling, myocardial oxygen-demand reduction, and endothelial risk-factor modification, whereas epicardial or microvascular spasm requires anti-vasoconstrictive therapy, particularly calcium-channel blockers. Mitochondrial biology is therefore used in this review to explain susceptibility and therapeutic hypotheses, not to replace ICFT-based endotyping or guideline-based management [1,3,11,12,14,15,16,17,18,19,20].

Despite this progress, most existing reviews on CMD focus primarily on clinical presentation, coronary physiology assessment, and management algorithms. Conversely, reviews of mitochondrial dysfunction in cardiovascular disease typically address cardiomyocyte biology, heart failure, and myocardial infarction, without engaging in sufficient depth with the coronary microvascular endothelium. Based on a review of current literature, the intersection of endothelial mitochondrial biology, CMD endotypes, and translational clinical implications has received limited focused attention.

The coronary microvascular endothelial cell occupies a uniquely vulnerable position at the interface of metabolic signaling, hemodynamic forces, inflammatory stimuli, and vasomotor regulation. While endothelial cells rely predominantly on glycolysis for ATP production, mitochondria in these cells serve functions that extend well beyond bioenergetics: they regulate redox tone, calcium signaling, inflammatory activation, oxygen sensing, and mitochondrial quality surveillance [21,22]. Perturbation of these mitochondrial functions, arising from cardiometabolic risk factors, aging, and sex-specific hormonal transitions, may amplify the impairments in nitric oxide (NO) bioavailability, endothelium-dependent hyperpolarization (EDH), and vascular barrier integrity that collectively define CMD.

This review synthesizes the mechanistic and clinical evidence linking endothelial mitochondrial dysfunction to CMD in INOCA, with emphasis on redox signaling, NO and EDH, sex-specific vulnerability, biomarkers, coronary function testing, and therapeutic implications. The goal is neither to present endothelial mitochondria as a universal explanation for INOCA nor to reduce CMD to a mitochondrial disorder, but to articulate the mechanistic connections with appropriate nuance and to define the translational opportunities that a mitochondrial perspective may offer. Box 1 summarizes the clinically established, biologically plausible, and still investigational aspects of endothelial mitochondrial dysfunction in CMD.

Box 1 **Key messages for clinicians and translational researchers.**

  Clinically established: INOCA/ANOCA and CMD are heterogeneous syndromes, and validated physiological testing identifies endotypes such as reduced CFR/MFR, elevated microvascular resistance, endothelial dysfunction, and epicardial or microvascular spasm. Endotype-guided management improves symptoms and quality of life, although hard-outcome data remain limited.

  Biologically plausible: Endothelial mitochondrial dysfunction may amplify CMD through mtROS generation, impaired NO bioavailability, eNOS uncoupling, disrupted EDH signaling, calcium stress, defective mitophagy, mtDNA-mediated inflammatory activation, endothelial apoptosis, and microvascular rarefaction.

  Still investigational: Circulating cf-mtDNA, mtDNA copy number, endothelial extracellular vesicles, mitochondrial-derived vesicles, and mitochondria-targeted therapies are not validated diagnostic, prognostic, or therapeutic tools for CMD in routine clinical care.

These messages synthesize clinical guideline/consensus and trial evidence [1,3,11,12,13,14,15,16,17,23], mechanistic vascular biology [18,19,24,25,26,27,28,29,30,31,32,33,34,35,36,37,38,39,40], and biomarker literature [18,19,20,41,42].

## 2. Materials and Methods

This narrative review was not designed as a systematic review or meta-analysis. Evidence was identified through a structured, non-exhaustive search aimed at capturing relevant clinical guidelines, expert consensus documents, landmark trials, prospective cohorts, mechanistic studies, and high-quality reviews on endothelial mitochondrial biology and CMD in ANOCA/INOCA.

Searches were performed in PubMed/MEDLINE and Google Scholar and supplemented by manual review of key reference lists and official ESC, ACC/AHA, and EAPCI guideline or consensus documents. The primary search period covered January 2000 to June 2026, with priority given to publications from 2020 onward. Earlier studies were included when foundational to endothelium-dependent hyperpolarization, endothelial mitochondrial signaling, nitric oxide biology, or endothelial nitric oxide synthase uncoupling.

Search terms combined: “INOCA,” “ANOCA,” “coronary microvascular dysfunction,” “microvascular angina,” “coronary function testing,” “invasive coronary function testing,” “coronary flow reserve,” “index of microvascular resistance,” “acetylcholine provocation,” “endothelial mitochondria,” “endothelial mitochondrial dysfunction,” “mitochondrial reactive oxygen species,” “mitochondrial quality control,” “mitophagy,” “nitric oxide,” “endothelium-dependent hyperpolarization,” “hydrogen peroxide,” “sex differences,” “women,” “menopause,” “estrogen signaling,” “CorMicA,” “CorCMR,” “WARRIOR,” and “PRIZE zibotentan.”

Priority was given to primary trial reports, guideline documents, registries, randomized trials, and mechanistic studies directly relevant to coronary or endothelial vascular biology. Reviews were used for synthesis where appropriate. Non-peer-reviewed sources, promotional supplements, methodologically unclear studies, and non-coronary microvascular studies were excluded unless directly relevant and interpreted cautiously. Formal screening, duplicate removal, risk-of-bias assessment, certainty grading, and PRISMA-style reporting were not performed. The review was prepared with attention to the Scale for the Assessment of Narrative Review Articles (SANRA), particularly regarding justification of the article’s importance, clarity of aims, description of the literature search, referencing, scientific reasoning, and appropriate presentation of evidence [43].

## 3. Clinical Spectrum, Endotypes, and Prognostic Implications of INOCA/CMD

### 3.1. Defining INOCA, ANOCA, and CMD

INOCA and ANOCA are often used interchangeably, although they have distinct diagnostic meanings. INOCA refers to ischemia with non-obstructive coronary arteries and requires objective evidence of myocardial ischemia together with the absence of obstructive epicardial coronary artery disease, usually defined as no stenosis ≥ 50% and no flow-limiting lesion. ANOCA is a broader anatomical descriptor that includes patients with angina and non-obstructive coronary arteries, regardless of whether ischemia has been formally documented. The EAPCI consensus framework defines CMD as one functional mechanism within this spectrum, alongside epicardial vasospastic angina, microvascular spasm, and mixed vasomotor dysfunction [3].

CMD should therefore not be considered synonymous with INOCA, but rather as a structural or functional abnormality of the coronary microcirculation that impairs regulation of coronary blood flow within the broader INOCA/ANOCA spectrum [3,14].

### 3.2. CMD Endotypes

CMD classification recognizes overlapping endotypes with different pathophysiological and therapeutic implications. Reduced CFR reflects the ratio of hyperemic to resting coronary blood flow and provides an integrated measure of microvascular vasodilatory capacity. A CFR below 2.0 or 2.5, depending on method and context, is generally considered abnormal and may result from increased resting flow, impaired hyperemic flow, or both [14,15].

Another important endotype is elevated microvascular resistance, assessed by indices such as the index of microvascular resistance (IMR) or hyperemic microvascular resistance (HMR), reflects impaired flow during maximal hyperemia; IMR > 25 units is commonly used as an abnormal threshold, although cut-offs vary [14,15]. Elevated microvascular resistance may reflect structural remodeling of the microcirculation, microvascular rarefaction, or functional vasoconstriction.

Microvascular spasm is identified when acetylcholine provocation produces ischemic symptoms, ischemic electrocardiographic changes, or impaired coronary flow without epicardial spasm, whereas endothelial dysfunction is assessed by abnormal endothelium-dependent vasomotor responses during acetylcholine infusion [3,11,14].

In clinical practice, these endotypes frequently overlap. Many patients demonstrate combinations of reduced CFR, elevated microvascular resistance, endothelial dysfunction, and abnormal vasomotor reactivity. This overlap highlights the heterogeneity of CMD and supports the need for mechanism-based assessment in both clinical research and routine care [3,8].

### 3.3. Limitations of Conventional Angiography and the Role of ICFT

Conventional coronary angiography visualizes the epicardial arteries and excludes obstructive CAD, but it does not assess the coronary microcirculation, which lies beyond the spatial resolution of angiographic imaging [14,15]. Therefore, a normal or near-normal coronary angiogram does not exclude CMD. Physiological testing is required to characterize the mechanisms underlying symptoms and ischemia in patients with suspected INOCA or ANOCA.

Invasive coronary function testing (ICFT) enables direct assessment of coronary microvascular and vasomotor function: adenosine-based testing evaluates CFR and IMR, whereas acetylcholine testing evaluates epicardial and microvascular vasomotor responses [3,14,15].

The 2024 ESC Guidelines strengthened the clinical role of ICFT by recommending coronary vasomotor assessment in patients with persistent angina and non-obstructive coronary arteries [1,2,15]. The CorMicA trial showed that ICFT-guided stratified therapy improved angina and quality of life at one year, while CorCMR demonstrated that non-invasive cardiac magnetic resonance (CMR)-based endotyping improved treatment appropriateness and patient-reported outcomes [11,12,13,18]. However, neither trial was powered to show reductions in hard cardiovascular outcomes [11,12,13,44].

### 3.4. Clinical Consequences and Prognosis

INOCA and CMD should not be described as uniformly benign, but their prognosis is also not uniformly severe. Patients often experience recurrent angina, impaired exercise tolerance, repeated healthcare encounters, and reduced quality of life [5,8,45]. The COVADIS international prospective cohort showed that persistent symptoms and clinically meaningful cardiovascular events occur in patients with microvascular angina [8].

Hard outcome data remain heterogeneous and appear to depend on diagnostic method and endotype. Meta-analytic evidence suggests that patients with microvascular angina experience MACE at a rate of 2.5 events per 100 patient-years (95% CI, 1.6–3.6), compared with 1.1 events per 100 patient-years (95% CI, 0.5–1.9) among patients with vasospastic angina [6]. In the Yale-CMD registry, CMD patients had higher adjusted MACE incidence than controls (adjusted incidence rate ratio, 3.8; 95% CI, 2.1–6.6) and higher time-to-first MACE risk (adjusted hazard ratio, 3.6; 95% CI, 1.2–10.7) [6]. Registry data from the Yale-CMD cohort similarly indicate that patients with CMD have an approximately fourfold higher risk of major adverse cardiovascular events compared with non-CMD controls [7]. Prospective endotype-stratified analyses also suggest that the microvascular dysfunction endotype carries the highest event burden among INOCA endotypes [46].

These findings support active diagnostic evaluation and individualized management, while avoiding uniform prognostic statements that ignore CMD heterogeneity and comorbidities such as diabetes, hypertension, obesity, and chronic kidney disease.

## 4. Endothelial and Mitochondrial Control of Coronary Microvascular Function

### 4.1. Structural and Functional Organization of the Coronary Microcirculation

The coronary microcirculation comprises arterioles measuring approximately 100–500 µm, pre-arterioles, with intramural arterioles < 100 µm, capillaries, and venules, and accounts for more than 70% of total coronary vascular resistance. Endothelial regulation of arteriolar tone is central to matching myocardial oxygen delivery with metabolic demand and depends on integration of shear stress, metabolic byproducts, neurohumoral mediators, inflammatory cytokines, and paracrine signals from vascular smooth muscle cells and cardiomyocytes [47,48].

The endothelium converts these signals into vasomotor responses through balanced release of vasodilator and vasoconstrictor mediators. Nitric oxide (NO), generated by endothelial nitric oxide synthase (eNOS) in response to shear stress, receptor stimulation, and intracellular calcium signaling, activates soluble guanylate cyclase in vascular smooth muscle cells and promotes relaxation; it is the dominant endothelium-derived vasodilator in epicardial conduit arteries [24]. Prostacyclin (PGI_2_) also supports vasodilation and exerts antiplatelet effects through IP receptor activation, linking vasomotor regulation with local thrombo-inflammatory control [47].

In small coronary resistance arterioles, EDH often becomes increasingly important, particularly when NO bioavailability is reduced [24,25]. EDH-mediated dilation involves endothelial small- and intermediate-conductance calcium-activated potassium channels, myoendothelial gap junction signaling, and diffusible EDH factors, including epoxyeicosatrienoic acids and hydrogen peroxide (H_2_O_2_) [25,49].

In contrast, endothelin-1 is the principal endothelium-derived vasoconstrictor; by activating endothelin type A receptors on vascular smooth muscle cells, it promotes sustained vasoconstriction and has been implicated in microvascular angina, as reflected by the rationale for the PRIZE trial of the selective endothelin type A receptors antagonist zibotentan. Adverse events were more common with zibotentan, 60.2%, compared with placebo (14.4%; *p* < 0.001) [50].

### 4.2. The Special Role of H_2_O_2_ as a Physiological EDH Factor in the Coronary Microcirculation

A key principle for interpreting mitochondrial oxidative stress in CMD is that reactive oxygen species (ROS) are not uniformly harmful. H_2_O_2_ can function either as a physiological signaling molecule or as a mediator of oxidative injury, depending on concentration, localization, and duration of exposure. A mouse coronary arteriole study identified eNOS-derived H_2_O_2_ as an endothelium-derived hyperpolarizing factor [51]. This concept was subsequently confirmed in human coronary arterioles, where H_2_O_2_ was identified as the primary transferable factor mediating flow-induced dilation [52].

Evidence from coronary resistance vessels indicates that NO predominates in larger conduit arteries, whereas H_2_O_2_-mediated EDH becomes increasingly important in smaller microvessels [25]. Mouse studies further showed that disruption of the physiological NO/EDH balance impairs cardiovascular homeostasis and that neuronal NO synthase-derived H_2_O_2_ contributes to coronary microcirculatory maintenance and diastolic function [53,54].

This distinction is central to CMD. Low, spatially restricted H_2_O_2_ supports physiological microvascular dilation, whereas diffuse and uncontrolled ROS production from dysfunctional mitochondria, uncoupled eNOS, or activated NADPH oxidases reduces NO bioavailability, disrupts the NO/EDH balance, and impairs vasodilator reserve. Therefore, indiscriminate antioxidant supplementation may fail to restore vasodilator function and could theoretically interfere with physiological EDH/H_2_O_2_ signaling [25].

### 4.3. Why Endothelial Mitochondria Matter Beyond ATP Production

Endothelial cells generate approximately 85% of ATP through glycolysis rather than mitochondrial oxidative phosphorylation [21,22]. This metabolic pattern suits their position at the blood–tissue interface, but it does not make mitochondria dispensable [26,27,28]. Instead, endothelial mitochondria primarily function as signaling organelles that regulate vascular homeostasis beyond ATP production [26].

Mitochondria generate superoxide and H_2_O_2_ at the electron transport chain, especially complexes I and III. At physiological levels, these ROS participate in redox signaling, eNOS regulation, vascular tone, and adaptive responses to hypoxia [26]. Endothelial mitochondria also shape intracellular calcium dynamics, influencing eNOS activation, endothelial permeability, and inflammatory signaling [27]. In addition, mitochondrial electron transport contributes to oxygen sensing through hypoxia-inducible signaling pathways that are sensitive to mitochondrial ROS gradients [26,27,28,29].

Mitochondrial dysfunction promotes endothelial inflammatory activation, including nuclear factor-κB signaling, and disrupts mitochondrial quality-control pathways such as fission, fusion, biogenesis, and mitophagy [30,31]. Failure of these systems may promote endothelial apoptosis, senescence, microvascular rarefaction, and loss of vascular integrity, partly through mitochondrial permeability transition pore opening and activation of mitochondria-dependent cell death pathways [26]. Overall, endothelial mitochondria are best understood as regulators of vascular tone, inflammation, cell survival, endothelial health, organ dysfunction, and aging, rather than as simple ATP-generating organelles [26,27,29].

## 5. Endothelial Mitochondrial Dysfunction as a Mechanistic Amplifier in CMD

**Figure 1:**
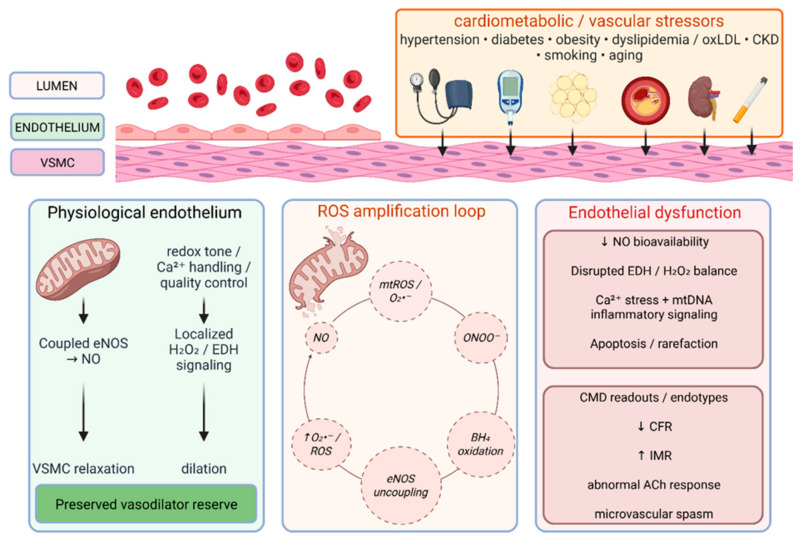
Endothelial mitochondrial redox signaling as a mechanistic amplifier in coronary microvascular dysfunction. This schematic provides an integrative overview of how cardiometabolic, vascular, aging-related, and sex-related stressors may converge on endothelial mitochondrial dysfunction. The central pathways include mtROS amplification, impaired NO bioavailability, eNOS uncoupling, disrupted NO/EDH balance, mitochondrial calcium stress, defective fission–fusion/mitophagy–biogenesis quality control, mtDNA-mediated inflammatory signaling, endothelial apoptosis, and microvascular rarefaction. Risk-factor-specific examples discussed in Section 6, including diabetes-related methylglyoxal/AR–cPLA2 signaling and dyslipidemia-related oxLDL–Nrf2/PPARγ regulation of mitochondrial fusion–fission balance, are represented conceptually as upstream mechanisms converging on this shared endothelial mitochondrial hub. These mechanisms may contribute to clinically measurable CMD/INOCA endotypes, including reduced CFR/MFR, elevated IMR/HMR, abnormal acetylcholine response, microvascular spasm, and impaired vasodilator reserve. The figure represents a mechanistic synthesis and should not be interpreted as evidence that current clinical tests directly measure endothelial mitochondrial injury. ACh, acetylcholine; BH_4_, tetrahydrobiopterin; CFR, coronary flow reserve; CKD, chronic kidney disease; CMD, coronary microvascular dysfunction; EDH, endothelium-dependent hyperpolarization; eNOS, endothelial nitric oxide synthase; H_2_O_2_, hydrogen peroxide; HMR, hyperemic microvascular resistance; IMR, index of microvascular resistance; MFR, myocardial flow reserve; mtDNA, mitochondrial DNA; mtROS, mitochondrial reactive oxygen species; NO, nitric oxide; oxLDL, oxidized low-density lipoprotein; PPARγ, peroxisome proliferator-activated receptor gamma; ROS, reactive oxygen species; VSMC, vascular smooth muscle cell. Created in BioRender. Kumric, M. (2026) https://BioRender.com/za6ho2p (accessed on 6 July 2026).

Most evidence linking endothelial mitochondrial dysfunction to CMD is mechanistic, derived from vascular biology studies, animal models, or ex vivo human microvascular preparations [26,27,28,29,30,31,32,33,55,56,57,58,59]. Therefore, mitochondrial abnormalities are best interpreted as plausible mechanistic amplifiers rather than clinically validated diagnostic or therapeutic targets in CMD [18,19]. Accordingly, the proposed amplification framework is summarized schematically in Figure 1, which should be read together with the pathway-level synthesis in Section 6. Figure 1 provides the integrated stressor–endothelial mitochondrial dysfunction–CMD endotype framework, whereas the detailed risk-factor-specific mechanisms, including diabetes-related methylglyoxal signaling and oxLDL–Nrf2/PPARγ–mitochondrial dynamics, are described below.

### 5.1. Mitochondrial ROS, NO Bioavailability, and eNOS Uncoupling

The interaction between mitochondrial ROS and NO bioavailability provides a mechanistic framework for impaired vasomotor regulation in CMD. Under cardiometabolic stress, excessive superoxide generated at mitochondrial complexes I and III reacts with NO to form peroxynitrite (ONOO^−^), reducing NO bioavailability and impairing vasodilation. Peroxynitrite oxidizes tetrahydrobiopterin (BH_4_), leading to eNOS uncoupling, in which eNOS produces superoxide rather than NO [24,34]. This creates a self-amplifying loop involving mitochondrial ROS, peroxynitrite formation, BH_4_ oxidation, eNOS uncoupling, further ROS accumulation, and impaired vasodilator reserve.

This cycle is not mitochondrial alone. NADPH oxidases, particularly NOX2 and NOX4, are major non-mitochondrial ROS sources that intersect with mitochondrial ROS signaling under cardiometabolic stress [34]. The eNOS–vascular oxidative stress axis is a critical determinant of endothelial function, and mitochondrial contributions to endothelial dysfunction interact with other ROS-producing systems to promote vascular injury and cardiovascular risk [24,28].

In the coronary microcirculation, reduced NO may increase reliance on EDH/H_2_O_2_-mediated dilation, but excessive mitochondrial ROS can also disrupt this compensatory pathway. A human and rat microvascular study reported that endothelial dynamin-related protein 1 (DRP1) overexpression shifted flow-mediated dilation from NO toward H_2_O_2_; in patients with CAD, this shift was associated with impaired dilation not fully rescued by antioxidant treatment [55]. These findings link mitochondrial fission dynamics to NO/EDH imbalance, although translation to ICFT-defined CMD endotypes remains incomplete.

### 5.2. Mitochondrial Calcium Handling and Endothelial Vasomotor Signaling

Calcium is a central second messenger for eNOS activation, and mitochondria actively shape intracellular calcium dynamics. Mitochondrial calcium uptake, mediated primarily through the mitochondrial calcium uniporter (MCU), regulates the amplitude and duration of cytosolic calcium signals [27]. Under mitochondrial stress, impaired calcium buffering may lead to cytosolic calcium overload, activation of pro-inflammatory pathways, disruption of endothelial barrier integrity, and promotion of vasoconstrictive signaling [27,29].

In the coronary microcirculation, calcium-dependent endothelial activation also contributes to EDH-mediated vasodilation. Cytosolic calcium activates endothelial small- and intermediate-conductance calcium-activated potassium channels, SKCa and IKCa, which promote endothelial hyperpolarization and subsequent electrical signaling to vascular smooth muscle cells (VSMCs) [54]. Mitochondrial calcium dysregulation may therefore impair both NO-dependent and EDH-dependent vasodilator mechanisms. In the context of cardiometabolic risk factors, mitochondrial calcium overload secondary to ROS-mediated activation of the mitochondrial permeability transition pore represents a proposed mechanistic pathway metabolic stress to endothelial apoptosis, microvascular rarefaction, and CMD [26,27].

### 5.3. Mitochondrial Dynamics, Mitophagy, and Biogenesis

Mitochondrial homeostasis depends on coordinated fission, fusion, mitophagy, and biogenesis [30,31,35]. DRP1-mediated fission helps isolate damaged mitochondrial segments; MFN1/2- and OPA1-mediated fusion supports network integrity; PINK1/Parkin-, FUNDC1-, and BNIP3-dependent mitophagy removes damaged mitochondria; and peroxisome proliferator-activated receptor gamma coactivator 1-alpha (PGC-1α)-mediated biogenesis replenishes the mitochondrial pool [30,35,36].

When this quality-control network is overwhelmed, damaged mitochondria accumulate and promote ROS generation, endothelial dysfunction, senescence, and apoptosis [29,31]. Impaired mitochondrial quality control is a major feature of cardiac microvascular endothelial injury, and mitochondrial quality surveillance has been identified as a key axis linking hyperglycemia to diabetes-related coronary microvascular endothelial dysfunction [30,31].

Excessive DRP1-mediated fission is particularly relevant in diabetic CMD models. Hyperglycemia promotes mitochondrial fragmentation in coronary microvascular endothelial cells, leading to disproportionate ROS generation, impaired eNOS coupling, inflammation, and apoptosis [30,31,56]. In diabetic myocardial microvascular injury, empagliflozin attenuated injury through AMPK-mediated inhibition of DRP1-dependent fission, and a related experimental study found activation of the AMPKα1/ULK1/FUNDC1 mitophagy pathway in coronary microvascular endothelial cell [56,57]. These experimental findings support mitochondrial quality control as a therapeutic hypothesis, but not as clinical evidence that empagliflozin improves CMD endotypes in INOCA.

Exercise provides a complementary preclinical example: in aged mice, exercise restored FUNDC1-dependent mitophagy through a PPARγ-mediated mechanism and protected against coronary endothelial senescence [58]. PGC-1α, a master regulator of mitochondrial biogenesis, is also upregulated by laminar shear stress, consistent with exercise-mediated endothelial benefit [36,59].

### 5.4. Mitochondrial DNA as an Innate Immune Signal

Mitochondrial DNA (mtDNA) contains unmethylated cytosine-phosphate-guanine dinucleotide motifs and becomes immunostimulatory when released into the cytoplasm or extracellular space. Damaged mitochondria may release mtDNA through mitochondrial permeability transition pore opening, outer membrane permeabilization, or mitochondrial-derived vesicle shedding [32,37].

Released mtDNA functions as a damage-associated molecular pattern (DAMP), an endogenous signal of cellular injury that activates innate immune receptors. Cytosolic mtDNA engages cyclic GMP-AMP synthase (cGAS), leading to cyclic GMP-AMP production and activation of the stimulator of interferon genes (STING) pathway, with subsequent type I interferon responses and NF-κB activation [33,37]. mtDNA can also activate TLR9, and oxidized mtDNA fragments may activate the NLRP3 inflammasome [32,37,38]. In retinal microvascular endothelial cells, pathological stimuli induced mtDNA release and cGAS-STING activation, promoting inflammatory endothelial injury [33].

Clinically, this pathway should be framed cautiously. Circulating cell-free mtDNA and mtDNA copy number are detectable in blood and have been associated with cardiovascular risk in exploratory studies [18,19]. However, they have not been validated as diagnostic or prognostic biomarkers specific to CMD or INOCA. At present, they should be considered mechanistically plausible research biomarkers requiring prospective validation against clearly defined CMD endotypes.

## 6. Cardiometabolic and Vascular Risk Factors Driving Endothelial Mitochondrial Dysfunction in CMD

Cardiometabolic and vascular risk factors impose mitochondrial stress through partially overlapping pathways that converge on endothelial dysfunction and impaired coronary microvascular regulation. As summarized in Figure 1, these mechanisms share common endothelial mitochondrial nodes and should be viewed as interconnected rather than mutually exclusive.

### 6.1. Diabetes and Insulin Resistance

Hyperglycemia is among the best-studied drivers of coronary microvascular endothelial cell mitochondrial dysfunction. Elevated glucose flux increases electron delivery to the respiratory chain, enhances superoxide generation, and exceeds mitochondrial antioxidant capacity [31,56,57]. In coronary microvascular endothelial cells, this promotes DRP1-dependent fission, impairs FUNDC1- and PINK1/Parkin-mediated mitophagy, suppresses PGC-1α-dependent biogenesis, and increases endothelial apoptosis [30,34,35,36,56,57]. A diabetes-focused mechanistic review identified mitochondrial quality surveillance as a central axis linking hyperglycemia to coronary microvascular endothelial dysfunction, with impaired mitophagy leading to accumulation of dysfunctional mitochondria, sustained ROS production, eNOS uncoupling, and reduced CFR [31]. Advanced glycation-related mediators, including methylglyoxal, may further promote CMD through direct coronary endothelial injury. In experimental coronary endothelial models, methylglyoxal downregulated androgen receptor signaling, increased ROS-dependent cytosolic phospholipase A2 activation, reduced anti-apoptotic Bcl-2 signaling, and promoted endothelial apoptosis and impaired coronary vasodilatory function [39]. This pathway provides a mechanistic link between diabetic carbonyl stress, sex-related endothelial vulnerability, and microvascular dysfunction, although human CMD-specific validation remains limited. Insulin resistance independently reduces eNOS activation through impaired PI3K/Akt signaling, thereby compounding NO deficiency [24,31].

### 6.2. Obesity and Systemic Inflammation

Visceral adiposity promotes chronic low-grade systemic inflammation characterized by increased circulating interleukin-6, tumor necrosis factor-alpha, and adverse adipokine signaling. These inflammatory mediators activate endothelial NF-κB, increase mitochondrial ROS generation, impair mitochondrial biogenesis, and promote endothelial insulin resistance [29,40,60]. Adipose-derived oxidative and inflammatory signals may also promote eNOS uncoupling and attenuate EDH-mediated vasodilation in the coronary microcirculation [40]. Sex differences in adipose distribution, menopausal transition, and risk-factor clustering may contribute to the higher CMD burden observed in women, but obesity alone should not be presented as a sufficient explanation for sex-specific CMD risk [60,61,62,63].

### 6.3. Hypertension and Mechanical Stress

Chronic hypertension exposes the coronary microvascular endothelium to increased transmural pressure, cyclic stretch, and disturbed shear stress. In contrast to laminar shear stress, which supports endothelial mitochondrial adaptation and PGC-1α signaling, disturbed shear stress promotes oxidase activation, mitochondrial fission, and ROS generation [28,40,59]. Angiotensin II and related neurohumoral signaling further increase mitochondrial oxidative stress, mitochondrial calcium overload, and eNOS uncoupling [24,27,29]. The resulting reduction in NO bioavailability, together with impaired EDH reserve, may contribute to increased microvascular resistance and reduced CFR in susceptible hypertensive patients [48,63].

### 6.4. Dyslipidemia and Oxidized LDL

Oxidized low-density lipoprotein (oxLDL) is directly injurious to endothelial cells and activates lectin-like oxLDL receptor-1 (LOX-1), which promotes mitochondrial dysfunction through increased ROS production, loss of mitochondrial membrane potential, and mitochondrial permeability transition pore opening [26,29,40]. OxLDL may also impair endothelial mitochondrial dynamics by disturbing the balance between mitochondrial fusion and fission. In human coronary artery endothelial cells, oxLDL-induced endothelial dysfunction has been linked to oxidative stress, reduced NO/eNOS signaling, apoptosis, and altered mitochondrial fusion–fission balance, whereas activation of the Nrf2/PPARγ pathway restored mitochondrial dynamics and improved endothelial function in experimental models [64]. These findings support the Nrf2–mitochondrial dynamics axis as a mechanistic bridge between dyslipidemia and endothelial mitochondrial injury, but it remains a preclinical pathway rather than a validated CMD-specific therapeutic target. Lipotoxicity from excess free fatty acids, particularly saturated fatty acids, may activate compensatory mitophagy; however, persistent lipid excess can overwhelm mitochondrial quality-control pathways and lead to accumulation of dysfunctional mitochondria [35,40]. Statins may preserve endothelial function not only by lowering LDL cholesterol, but also by reducing oxidative stress and improving endothelial vasomotor signaling [16,17]. However, direct evidence that lipid-lowering therapy restores endothelial mitochondrial function specifically in CMD remains limited.

### 6.5. Aging and Endothelial Senescence

With aging, endothelial mitochondria accumulate oxidative damage and show impaired mitophagy and biogenesis, promoting endothelial senescence characterized by reduced eNOS expression and activity, impaired eNOS/EDH signaling, and a pro-inflammatory and pro-thrombotic endothelial phenotype [28,29,58]. Rarefaction of the coronary microvascular network may partly reflect age-related endothelial apoptosis driven by mitochondrial dysfunction [40]. A vascular aging review highlighted age-related mitochondrial deterioration in endothelial cells as a major contributor to vascular aging and increased cardiovascular risk [28]. In preclinical coronary endothelial aging, FUNDC1 depletion has been linked to impaired mitophagy and endothelial senescence, whereas exercise restored FUNDC1-dependent mitophagy [58]. These age-related mechanisms may increase CMD vulnerability, particularly when they overlap with cardiometabolic risk clustering and postmenopausal hormonal changes, as discussed in Section 7 [60,62].

### 6.6. Chronic Kidney Disease and Uremic Stress

Chronic kidney disease exposes endothelial cells to uremic toxins, oxidative stress, chronic inflammation, and impaired NO signaling. Uremic solutes, including indoxyl sulfate, p-cresyl sulfate, and asymmetric dimethylarginine, are biologically plausible mediators of endothelial mitochondrial injury [40]. Asymmetric dimethylarginine directly inhibits eNOS activity and may contribute to eNOS uncoupling, thereby reducing NO bioavailability [24,34]. Uremic stress may also promote mitochondrial membrane disruption, mtDNA release, and DAMP-mediated innate immune activation [32,37,40]. Patients with chronic kidney disease frequently exhibit impaired coronary microvascular function, but the direct coronary endothelial mitochondrial evidence remains less mature than for diabetes mellitus, aging, and cardiometabolic inflammation [40,48].

### 6.7. Smoking and Environmental Stressors

Tobacco combustion products, including acrolein and other reactive aldehydes, damage mitochondrial electron transport-chain proteins and deplete glutathione, thereby amplifying mitochondrial ROS generation [28,40]. Smoking also reduces eNOS signaling and promotes oxidative inactivation of NO, favoring impaired endothelial vasodilator reserve [24,28]. These mechanisms plausibly contribute to CMD, particularly when smoking coexists with diabetes mellitus, hypertension, dyslipidemia, or chronic kidney disease. Although smoking cessation is associated with improved endothelial function, CMD-specific evidence linking cessation to restoration of endothelial mitochondrial quality control remains limited [16,40].

Table 1 details the specific endothelial mitochondrial mechanisms linking established risk factors to coronary microvascular dysfunction, providing a mechanistic framework for understanding how these factors converge at the mitochondrial level to drive disease.

## 7. Sex-Specific Determinants of CMD and INOCA

Sex influences CMD and INOCA through differences in disease recognition, hormonal signaling, endothelial redox biology, cardiometabolic risk clustering, symptom burden, and trial representation. CMD should therefore be discussed as a sex-influenced syndrome rather than a female-only condition, while still recognizing clinically relevant CMD phenotypes in men [60,61,62,63].

### 7.1. Sex Distribution of CMD/INOCA and the Risk of Framing Bias

INOCA and CMD are more frequently recognized in women than in men across consensus documents, registries, cohorts, and contemporary reviews [3,4,8,9]. In comparable INOCA populations, women undergoing ICFT show a higher prevalence of CMD, higher IMR, and lower CFR than men [61,65]. Women with microvascular angina also report greater symptom burden and poorer quality of life in cohorts with objectively documented CMD [3,8,61].

However, CMD should not be conceptualized as a female-only disorder. Men are not protected from CMD, and contributing mechanisms may differ by sex [62,63]. A cardiac magnetic resonance cohort found that traditional cardiovascular risk factors were more strongly associated with CMD in men, whereas cardiac structural parameters were more prominent contributors in women [62]. These findings argue against a single sex-neutral model and suggest that structural, metabolic, hormonal, and inflammatory mechanisms may contribute differently across sexes [62,63].

Clinically, framing INOCA as predominantly or exclusively female may delay coronary function testing in symptomatic men, whereas ignoring the higher observed burden in women risks under-recognition of sex-specific biology and symptom expression [3,62,63].

### 7.2. Sex-Specific Hormonal Mechanisms

Estrogen signaling is relevant to CMD because estrogen receptor activation supports eNOS through non-genomic PI3K/Akt and genomic pathways, promotes antioxidant gene expression, and attenuates endothelial inflammatory activation [60]. Experimental endothelial studies show that 17β-estradiol can rapidly activate eNOS through estrogen receptor-dependent mechanisms, including ERα-mediated and PI3K/Akt-dependent signaling [66,67,68]. These pathways support the plausibility that estrogen preserves endothelial NO bioavailability, although direct extrapolation to human coronary CMD requires caution.

The menopausal transition, characterized by declining estradiol, is associated with reduced endothelial function and increased oxidative stress [69]. In the coronary microcirculation, these changes may contribute to reduced NO bioavailability and impaired vasodilator reserve [60]. WISE data further show that CMD is common in women with chest pain without obstructive coronary disease, that years since menopause are associated with lower coronary flow velocity reserve, and that impaired coronary microvascular reactivity predicts adverse cardiovascular outcomes [70,71].

However, estrogen loss should not be presented as a single cause of CMD. Estrogen signaling is modified by age, receptor biology, oxidative receptor modification, adipose redistribution, inflammation, androgen balance, and cardiometabolic risk [60]. Menopause should therefore be framed as a vulnerability-modifying transition, and hormonal mechanisms should complement endotype-based assessment because CMD also occurs in men and premenopausal women through multiple risk pathways [3,60,61,62,63].

### 7.3. Mitochondrial Redox Signaling and Sex-Specific Vulnerability

Sex differences in mitochondrial biology may amplify CMD vulnerability after menopause. Estrogen has been linked to mitochondrial biogenesis through estrogen receptor beta (ERβ) and PGC-1α signaling, supporting mitochondrial density, antioxidant capacity, and endothelial resilience [60,72,73,74]. Both ERα- and ERβ-dependent pathways may influence mitochondrial function, although most evidence comes from experimental vascular or non-coronary endothelial models rather than human coronary microvascular endothelial cells [72,73].

Experimental and translational studies indicate that estrogen regulates mitochondrial function, respiratory-chain activity, biogenesis, and redox balance [72,73]. Estradiol improved mitochondrial efficiency and reduced oxidative stress in cerebral vessels and reduced mitochondrial superoxide in cultured human brain microvascular endothelial cells [74,75]. PGC-1α links sex hormone signaling to mitochondrial antioxidant defense and endothelial survival [36,76]. With reduced estrogen signaling, mitochondrial support may decline, favoring mitochondrial ROS accumulation, eNOS uncoupling, and impaired EDH [24,25,28,34,36,60].

Because coronary H_2_O_2_ can act as a physiological EDH mediator, the relevant abnormality is disrupted redox compartmentalization and NO/EDH balance, not simply excess ROS [25,51,52,53,54]. The androgen receptor (AR)–ROS–cytosolic phospholipase A2 pathway has also been implicated in experimental endothelial apoptosis and CMD, but human validation remains insufficient; it should be framed as plausible rather than established [39,60].

### 7.4. Quality of Life and Trial Design Implications

Women with INOCA and CMD frequently report higher angina burden and worse quality of life than men, even when CMD is objectively documented [3,8,45,61]. This disparity may reflect differences in symptom generation, pain processing, inflammatory sensitization, comorbidity patterns, psychosocial factors, or healthcare access. It may also indicate that conventional physiological indices, including CFR and IMR, do not fully capture sex-specific mechanisms of symptom expression [8,45,62].

Future CMD trials should incorporate sex as a prespecified biological variable rather than a secondary descriptive characteristic. Menopause status, age at menopause, hormonal history, and menopausal hormone therapy should be considered when biologically relevant [60]. WARRIOR, which enrolled symptomatic women with suspected INOCA/non-obstructive CAD, is an important sex-specific management study, but its findings require cautious interpretation in relation to CMD endotypes, background therapy, and generalizability to men [77]. Trials of mitochondria-targeted or endothelial-targeted therapies should include adequate representation of both sexes, prespecified sex-stratified analyses, and sufficient power to test biologically plausible sex-specific treatment effects [60,61,77].

## 8. Diagnostic Evaluation: Coronary Function Testing and Translational Biomarkers

### 8.1. Clinical Diagnostic Pathway

Evaluation of suspected INOCA begins with exclusion of obstructive CAD by coronary computed tomography angiography or invasive angiography [1,3,10,15]. Coronary computed tomography angiography has high negative predictive value and defines atherosclerotic burden, but it does not assess coronary microvascular function [1,10,15]. Persistent angina or objective ischemia after a normal or non-obstructive angiogram should therefore prompt functional assessment. Standardized microvascular angina criteria require ischemic symptoms, absence of obstructive CAD, objective ischemia, and evidence of impaired microvascular function, such as reduced CFR or inducible microvascular spasm [78].

Noninvasive CMD assessment includes positron emission tomography (PET), stress cardiac magnetic resonance, and transthoracic Doppler echocardiography. PET myocardial perfusion imaging with quantitative myocardial blood flow and myocardial flow reserve (MFR) is the best validated noninvasive method for quantifying impaired CFR, enables absolute myocardial blood flow assessment, and is less susceptible to attenuation artifacts than SPECT [15,45,79]. Stress CMR provides high-resolution semiquantitative or quantitative perfusion assessment and has been incorporated into CMD pathways, including CorCMR [13,15,44,80,81].

Transthoracic Doppler echocardiography can measure coronary flow velocity reserve, most often in the left anterior descending coronary artery, and iPOWER trial supports its feasibility and prognostic relevance in women with angina, although operator dependency and limited spatial coverage remain constraints [15,45,82,83].

ICFT remains the reference standard for CMD characterization and endotype assignment [3,14,15]. Comprehensive protocols typically include acetylcholine provocation, adenosine-based CFR assessment, IMR, and resistance indices such as hyperemic microvascular resistance or microvascular resistance reserve [14,15]. ICFT distinguishes endothelial dysfunction, impaired vasodilator reserve, elevated microvascular resistance, epicardial spasm, microvascular spasm, and mixed phenotypes, which is clinically relevant because treatment differs across endotypes [1,3,14,15].

Acetylcholine testing evaluates endothelium-dependent vasomotor function. Epicardial spasm is generally defined by symptoms, ischemic electrocardiographic changes, and at least 90% epicardial diameter reduction, whereas microvascular spasm is suggested by symptoms and ECG changes without epicardial spasm [3,14,15]. Acetylcholine normally promotes endothelial NO-mediated vasodilation, but endothelial dysfunction may produce paradoxical vasoconstriction or spasm [84].

Adenosine-based CFR reflects maximal hyperemic to resting coronary flow. CFR values of 2.0–2.5 commonly define impaired vasodilator capacity, and IMR >25 U commonly defines elevated microvascular resistance [14,15]. Because CFR reflects both resting and hyperemic flow, reduced CFR may result from increased resting flow, impaired hyperemia, remodeling, vasoconstriction, diffuse non-obstructive atherosclerosis, extravascular compression, or combined mechanisms [78,79,85]. Therefore, abnormal CFR/MFR and acetylcholine responses should be interpreted as physiological and vasomotor phenotypes, not direct markers of endothelial mitochondrial injury. Mitochondrial dysfunction remains a mechanistic amplifier requiring biomarker or translational evidence [14,15].

A practical endotype-guided framework linking diagnostic readouts, pathophysiological interpretation, and potential therapeutic approaches is summarized in Table 2.

### 8.2. Investigational Mitochondrial Biomarkers and Complementary Clinical Biomarkers

At present, no mitochondrial biomarker is currently validated as a diagnostic or prognostic tool for CMD. Candidate biomarkers have mechanistic rationale but remain investigational and should not be used as clinical diagnostic tests [18,19,20].

Circulating cell-free mitochondrial DNA (cf-mtDNA) has been associated with cardiovascular risk and inflammatory activation, and leukocyte mtDNA copy number has been associated with endothelial dysfunction measured by flow-mediated dilation. However, cf-mtDNA and mtDNA-copy number are affected by assay conditions, sample handling, platelet contamination, DNA extraction, age, exercise, renal function, systemic inflammation, and acute tissue injury [18,19]. Leukocyte mtDNA-copy number also does not necessarily reflect mitochondrial content or function in coronary endothelial cells [19].

Clinically available biomarkers should be clearly distinguished from mitochondrial-specific candidates. BNP or NT-proBNP, blood glucose or HbA1c, renal indices, and inflammatory markers may identify cardiometabolic, renal, or myocardial-stress phenotypes that correlate with impaired CFR/MFR, but they do not demonstrate endothelial mitochondrial injury. In an exploratory study using invasive coronary physiological assessment, BNP and casual blood glucose showed diagnostic signal for CFR < 2.0, and their combination improved discrimination for CFR impairment; however, these markers should be interpreted as complementary clinical risk markers rather than CMD-specific mitochondrial biomarkers [86].

Oxidative stress markers provide plausible but nonspecific redox readouts. Malondialdehyde has shown discriminatory value for CAD severity in some cohorts, while 8-hydroxy-2′-deoxyguanosine, reduced glutathione, and glutathione peroxidase-related measures may reflect oxidative injury but are not validated for CMD endotyping [45,87]. Their limitation is that systemic oxidative stress does not identify the vascular bed, cell type, or CMD mechanism responsible.

Extracellular vesicles may support endothelial and mitochondrial biomarker discovery because they can carry mitochondrial cargo, including mtDNA, and mitochondrial-derived vesicles may contribute to cardiovascular EV biology [41,42]. Endothelial-enriched EV profiling using CD31, CD62E, CD105, or CD144 may indicate endothelial activation or injury, but CMD thresholds are lacking [20,41,42].

Metabolomics and acylcarnitine profiling may provide insight into mitochondrial substrate handling and beta-oxidation stress, although CMD signatures remain exploratory and require prospective validation in rigorously defined endotypes [21,22,45]. Inflammatory markers such as interleukin-6 and high-sensitivity C-reactive protein have been associated with CMD and reduced CFR, but cannot distinguish mitochondrial injury from other inflammatory drivers [45].

Endothelial colony-forming cells provide an ex vivo platform for studying patient-derived endothelial mitochondrial function, but this approach remains translational rather than clinically deployable [20]. Overall, mitochondrial and endothelial biomarkers lack CMD-specific validation, tissue specificity, and preanalytical standardization. The most appropriate strategy is prospective integration of ICFT-defined CMD endotypes with circulating mitochondrial, endothelial, metabolic, and inflammatory biomarkers [14,15,18,19,20,45].

## 9. Endotype-Guided Management and Emerging Mitochondrial Therapeutic Opportunities in CMD/INOCA

### 9.1. Endotype-Guided Therapy as the Current Treatment Framework

Treatment for INOCA and CMD should be guided by the dominant coronary vasomotor endotype rather than applied uniformly across the heterogeneous INOCA spectrum. For clarity, the therapeutic discussion below separates current endotype-guided and guideline/consensus-supported approaches from mitochondria-targeted or cardiometabolic strategies that remain mechanistically plausible but insufficiently validated as CMD-specific therapies. This approach is supported by EAPCI consensus guidance, the 2024 ESC chronic coronary syndrome guidelines, and CorMicA and CorCMR, which showed that diagnostic endotyping linked to targeted therapy improves angina-related outcomes and quality of life [1,3,11,12,13]. Observational data also suggest that physiological endotype contributes to prognostic stratification in non-obstructive CAD [46].

The key distinction is between structural microvascular dysfunction, characterized by reduced CFR and/or increased microvascular resistance, and vasomotor disorders, including epicardial or microvascular spasm [3,14,15]. Reduced CFR or elevated resistance phenotypes may benefit from therapies that reduce myocardial oxygen demand, improve endothelial function, or modify cardiometabolic risk, whereas spasm-predominant phenotypes require anti-vasoconstrictive therapy, particularly calcium channel blockers [1,3,16,17,23].

Endotype-guided care does not imply that mitochondrial mechanisms can currently be measured or directly targeted in routine practice; rather, endothelial mitochondrial biology provides a framework for understanding why exercise, risk-factor control, endothelial protection, and future mitochondrial strategies may be relevant in selected CMD phenotypes [20,28,40,56,57,58].

### 9.2. Guideline-Based and Clinically Supported Therapies

Lifestyle modification and risk-factor management remain the foundation of CMD treatment. Exercise, weight management, smoking cessation, blood-pressure control, lipid-lowering therapy, and diabetes management target upstream drivers of endothelial mitochondrial dysfunction, including oxidative stress, inflammation, impaired NO signaling, mitochondrial fission, defective mitophagy, and reduced biogenesis [1,3,16,40]. Among lifestyle interventions, smoking cessation and exercise training have the strongest support for improving endothelial and coronary vascular health [16,88]. Cardiac rehabilitation and supervised exercise are also relevant in INOCA and are mechanistically linked to endothelial mitochondrial biogenesis, PGC-1α signaling, FUNDC1-dependent mitophagy, and NO/EDH balance [5,58,59,89,90].

Renin–angiotensin system inhibitors and statins are recommended for risk-factor management and may improve endothelial function through reduced oxidative stress, improved eNOS coupling, and attenuation of renin–angiotensin-mediated vascular injury, but they should not be presented as proven mitochondrial therapies for CMD [1,3,16,17,24]. For reduced CFR or elevated resistance phenotypes, beta-blockers may reduce symptoms by lowering heart rate, myocardial oxygen demand, and improving diastolic perfusion time [3,16,17,23]. Ranolazine and nicorandil may be considered in selected symptomatic patients, with ranolazine targeting late sodium current and nicorandil providing nitrate-like and ATP-sensitive potassium-channel effects; however, hard outcome data in CMD are lacking [17,23,91,92]. In vasospastic or microvascular spasm phenotypes, calcium channel blockers are the mainstay, with long-acting nitrates or nicorandil as selected adjuncts; beta-blockers should be used cautiously or avoided in spasm-predominant disease [1,3,17,23].

### 9.3. Landmark Trials and Interpretation of Evidence

CorMicA showed that ICFT-guided stratified therapy improves angina and quality of life but was not powered for myocardial infarction, mortality, or other hard endpoints [11,12].

CorCMR similarly supports noninvasive CMR-based endotyping for diagnostic reclassification and patient-reported benefit, not proven event reduction [13,44].

WARRIOR tested intensive statin, ACE inhibitor/ARB, and low-dose aspirin therapy versus usual care in symptomatic women with suspected INOCA/non-obstructive CAD and did not significantly reduce the five-year composite endpoint. At 2.5 years, the primary outcome did not differ significantly between intensive medical therapy and usual care (17.84% vs. 16.17%; unadjusted site-stratified HR, 1.13; 95% CI, 0.94–1.37; *p* = 0.2009). This result should be interpreted in the context of endotype heterogeneity, medication crossover, usual-care contamination, pandemic-related under-enrollment, and lack of CMD endotype enrichment [77].

PRIZE tested zibotentan in microvascular angina and found no improvement in exercise duration or angina symptoms; this does not exclude endothelin-1 biology in CMD but indicates that this specific strategy, dose, duration, and population did not translate into clinical benefit [50].

### 9.4. Mitochondria-Targeted Therapeutic Windows

The therapeutic implications of endothelial mitochondrial biology should be framed as translational opportunities, not immediate clinical prescriptions. Accordingly, SGLT2 inhibitors, GLP-1 receptor agonists, mitochondria-targeted antioxidants, and direct modulators of fission, mitophagy, or innate immune signaling should be presented as hypothesis-generating for INOCA/CMD unless tested in endotype-defined CMD trials. No mitochondria-targeted drug is validated as CMD-specific therapy in endotype-defined INOCA. The current value of mitochondrial biology lies in mechanistically enriched trial design, patient selection, and biomarker or physiological endpoints capable of testing whether mitochondrial modulation improves coronary microvascular function [16,18,19,20,28,40,56,57,58,59].

Sodium–glucose cotransporter 2 (SGLT2) inhibitors are promising from a mitochondrial perspective because empagliflozin protects coronary microvascular endothelial cells in preclinical models through AMPK-mediated inhibition of DRP1-dependent fission and activation of AMPKα1/ULK1/FUNDC1 mitophagy [56,57]. Clinical outcome trials support cardiovascular and cardiorenal benefit in diabetes, heart failure, chronic kidney disease, and elevated cardiovascular risk, and mechanistic analyses suggest mitochondrial quality-control effects [93,94,95]. A clinical mechanistic study also reported improved endothelial function and reduced mitochondrial oxidative stress with empagliflozin in frail hypertensive and diabetic patients [96]. However, these data do not prove CMD-specific benefit in INOCA.

Glucagon-like peptide-1 (GLP-1) receptor agonists also have mechanistic plausibility through improved NO bioavailability, reduced oxidative stress, and attenuation of endothelial inflammation [97]. In a large-animal chronic ischemia model, semaglutide improved myocardial perfusion and function through AMPK pathway activation [98]. These findings support the hypothesis that GLP-1 receptor agonists could influence coronary microvascular function, especially in obesity, diabetes, or insulin resistance. However, human CMD-specific trial data remain insufficient, and these agents should not be presented as established CMD therapies [97,98].

Mitochondria-targeted antioxidants, Coenzyme Q10, NAD+ precursor strategies, AMPK/sirtuin/PGC-1α/DRP1/mitophagy modulation, and anti-inflammatory approaches targeting cGAS-STING, TLR9, or NLRP3 remain investigational or preclinical for CMD [20,28,33,35,36,37,38,56,57]. Near-term progress will require endotype-enriched trials combining coronary physiological endpoints, symptoms, quality-of-life measures, and validated endothelial or mitochondrial biomarkers [14,15,18,19,20,45]. Table 3 outlines therapeutic windows for targeting endothelial mitochondrial dysfunction in CMD/INOCA, translating the pathophysiological mechanisms into actionable clinical strategies across guideline-supported therapies to emerging mitochondria-targeted approaches.

## 10. Future Directions

Future CMD/INOCA research should move beyond broad diagnostic labels and require endotype-defined enrollment. ICFT-documented CMD should be preferred; isolated vasospastic angina, structural myocardial disease, and patients without demonstrable coronary physiological abnormality should be excluded or analyzed as prespecified strata. When ICFT is not feasible, PET-derived MFR or quantitative stress CMR may support screening or follow-up, but imaging-defined CMD should not be pooled uncritically with ICFT-defined CMD [3,9,13,46,78,79,80,81].

Mechanistic trials should integrate coronary physiology with standardized mitochondrial and endothelial biomarker collection. Candidate panels may include cf-mtDNA, mtDNA-copy number, oxidative stress markers, endothelial EVs, mitochondrial-derived vesicles, inflammatory markers, and patient-derived endothelial colony-forming cells, sampled at baseline and after intervention. Validation should test associations with CFR, IMR, microvascular resistance reserve, HMR, and acetylcholine response, using standardized microvascular angina criteria and rigorous preanalytical control for sample handling, renal function, inflammation, recent exercise, and medication use [14,15,18,20,41,42,78,79,85,87].

Sex and hormonal biology should be incorporated prospectively. Trials should prespecify sex-stratified analyses, enroll adequate numbers of women and men, and collect menopausal status, years since menopause, hormone therapy exposure, sex hormone levels where feasible, and cardiometabolic risk clustering. WARRIOR highlights the limitations of sex-specific trials that are not endotype-defined [3,8,61,62,66,67,68,69,70,71,72,73,74,75,76].

Endpoints should combine coronary physiology, patient-reported outcomes, functional capacity, imaging, and exploratory biomarker panels. CFR, MFR, IMR, microvascular resistance reserve, and acetylcholine response should be interpreted as physiological or vasomotor endpoints, not direct mitochondrial readouts. Single circulating mitochondrial biomarkers should not serve as primary endpoints until CMD-specific thresholds are validated [13,14,15,18,19,20,41,42,45,78,79,85,87].

The central priority is an endotype-enriched, sex-informed, biomarker-standardized trial strategy capable of testing whether mitochondrial modulation improves CMD physiology, symptoms, and quality of life [11,12,46,77].

## 11. Conclusions

Coronary microvascular dysfunction within the INOCA/ANOCA spectrum represents a heterogeneous but clinically significant phenotype associated with recurrent angina, reduced quality of life, and increased cardiovascular risk when objectively confirmed. This heterogeneity is not a limitation but a defining feature that should guide diagnosis, risk stratification, and therapy. Endothelial mitochondria provide a unifying mechanistic framework, regulating redox signaling, calcium handling, inflammatory activation, mitophagy, endothelial survival, and the balance between nitric oxide and endothelium-dependent hyperpolarization. Cardiometabolic stressors, aging, chronic kidney disease, and postmenopausal hormonal changes may converge on these pathways, contributing to CMD susceptibility, while sex-specific biology modifies risk without restricting CMD to women.

Current evidence is strongest in preclinical and cardiometabolic settings, particularly diabetes, whereas direct human evidence linking endothelial mitochondrial dysfunction causally to ICFT-defined CMD remains limited. Accordingly, abnormal acetylcholine responses, reduced coronary flow reserve, and reduced myocardial flow reserve should be interpreted as validated physiological phenotypes rather than direct markers of mitochondrial dysfunction, and circulating mitochondrial biomarkers remain investigational. Clinical management should remain endotype-guided, integrating anatomical, ischemic, noninvasive, and invasive functional assessment. Lifestyle interventions, risk-factor modification, and guideline-directed antianginal therapies remain foundational, while mitochondria-targeted strategies and emerging cardiometabolic drugs require CMD-specific validation. Future progress will depend on endotype-enriched, sex-informed studies integrating coronary physiology with validated mitochondrial biomarkers.

## Figures and Tables

**Table 1:** Endothelial mitochondrial mechanisms potentially linking risk factors to coronary microvascular dysfunction.

Mitochondrial/Endothelial Process	Effect on Coronary Microvascular Physiology	Clinical or Research Correlate	Evidence Maturity
Mitochondrial ROS/NO imbalance	Reduced NO bioavailability; impaired vasodilator reserve	Reduced CFR; endothelial dysfunction on ACh testing	Established vascular mechanism [24,28,34,40]
eNOS uncoupling/peroxynitrite	Superoxide production by eNOS; BH_4_ depletion loop	Reduced CFR; elevated IMR in diabetes and hypertension	Established vascular mechanism [24,31,34,40]
Impaired EDH/H_2_O_2_ signaling	Loss of compensatory microvascular vasodilation	Reduced flow-mediated dilation; diastolic dysfunction	Preclinical CMD evidence [25,51,52,53,54]
Mitochondrial calcium stress	Cytosolic Ca^2+^ overload; endothelial activation and barrier disruption	Endothelial dysfunction; inflammation markers	Preclinical CMD evidence [26,27,29,40]
Excessive fission/impaired fusion	Mitochondrial fragmentation; amplified ROS; impaired respiratory signaling	Microvascular injury in diabetic CMD models	Preclinical CMD evidence [30,31,55,56]
Defective mitophagy/biogenesis	Accumulation of dysfunctional mitochondria; endothelial apoptosis/senescence	Microvascular rarefaction; coronary flow impairment	Preclinical CMD evidence [31,35,36,56,57,58]
mtDNA release/innate immune activation	cGAS-STING/TLR9/NLRP3 activation; sterile inflammation	Circulating cf-mtDNA (research biomarker only)	Research biomarker only [18,19,32,33,37,38,41,42]
Endothelial senescence/apoptosis	Microvascular rarefaction; impaired vasodilator capacity	Age-related reduced CFR; endothelial dysfunction	Emerging human evidence [28,29,58,60,62]

Abbreviations: ACh, acetylcholine; BH4, tetrahydrobiopterin; cf-mtDNA, circulating cell-free mitochondrial DNA; CFR, coronary flow reserve; CMD, coronary microvascular dysfunction; EDH, endothelium-dependent hyperpolarization; eNOS, endothelial nitric oxide synthase; IMR, index of microvascular resistance; NO, nitric oxide; ROS, reactive oxygen species.

**Table 2:** Endotype-guided framework linking coronary function testing readouts with clinical implications in INOCA/ANOCA and CMD.

Dominant Endotype	Main Diagnostic Readout	Clinical Implication
Reduced CFR/MFR or elevated IMR/HMR [1,3,14,15,16,17]	PET/CMR/TTDE or ICFT	Impaired vasodilator reserve or increased resistance; prioritize risk-factor control, endothelial protection, and antianginal therapy selected for non-spasm phenotypes.
Epicardial or microvascular spasm [1,3,14,15,17,23]	ACh provocation	Vasomotor hyperreactivity; prioritize calcium-channel blockers, with nitrates or nicorandil as selected adjuncts.
Mixed CMD/vasomotor dysfunction [1,3,11,12,14,15,16,17,23]	Combined abnormal CFR/MFR, IMR/HMR, or ACh response	Overlapping mechanisms; use combined endotype-guided therapy and reassess symptoms, ischemia, and risk-factor control.

Abbreviations: ACh, acetylcholine; CFR, coronary flow reserve; CMD, coronary microvascular dysfunction; CMR, cardiac magnetic resonance; HMR, hyperemic microvascular resistance; ICFT, invasive coronary function testing; IMR, index of microvascular resistance; MFR, myocardial flow reserve; PET, positron emission tomography; TTDE, transthoracic Doppler echocardiography.

**Table 3:** Therapeutic windows for endothelial mitochondrial dysfunction in CMD/INOCA.

Intervention Class	Intended Mechanistic Target	Clinical Readiness	Key Caveat	Key References
Exercise/cardiac rehabilitation	Mitochondrial biogenesis; FUNDC1 mitophagy; NO/EDH balance; shear-mediated endothelial remodeling	Guideline/consensus-supported for selected endotypes	Most evidence from mixed CAD populations; INOCA-specific data expanding	[5,58,59,88,89,90]
ACE inhibitor/ARB/statin-based endothelial risk modification	Oxidative stress reduction; eNOS recoupling; renin–angiotensin suppression	Guideline/consensus-supported for selected endotypes	Evidence from risk factor populations; CMD-specific hard outcome data lacking	[1,3,16,17,24]
Calcium channel blockers for spasm-predominant disease	VSMC relaxation; prevention of coronary vasospasm	Guideline/consensus-supported for selected endotypes	Benefit greatest in vasospastic CMD; may be less effective as monotherapy for structural CMD without spasm	[1,3,17,23]
Beta-blockers for selected reduced CFR/high-resistance phenotypes	Reduced myocardial oxygen demand; prolonged diastolic filling	Clinically plausible but indirect evidence	Potentially harmful in vasospastic-predominant phenotypes	[3,16,17,23]
Ranolazine/nicorandil where appropriate	Late Na^+^ channel inhibition; ATP-sensitive K^+^ channel opening; mitophagy modulation (nicorandil)	Clinically plausible but indirect evidence	Preclinical mitophagy data for nicorandil; no hard outcome trials	[17,23,91,92]
SGLT2 inhibitors/GLP-1 receptor agonists	Mitochondrial quality control (AMPK/DRP1/mitophagy); endothelial NO/ROS balance	Clinically plausible but indirect evidence	Benefits established in cardiometabolic disease; CMD-specific trial data absent	[56,57,93,96,97,98]
Mitochondria-targeted antioxidants (mitoquinone, elamipretide)	Mitochondrial ROS scavenging; eNOS recoupling	Preclinical/early translational	No CMD-specific clinical trial data; no approved indication	[20,28]
Mitophagy/fission/cGAS-STING/NLRP3-directed approaches	Mitochondrial quality surveillance; innate immune suppression	Not ready for routine care	Exclusively preclinical or early mechanistic evidence	[20,33,35,36,37,38,56,57]

Abbreviations: ACE, angiotensin-converting enzyme; AMPK, AMP-activated protein kinase; ARB, angiotensin receptor blocker; ATP, adenosine triphosphate; CFR, coronary flow reserve; cGAS-STING, cyclic GMP-AMP synthase-stimulator of interferon genes; CMD, coronary microvascular dysfunction; DRP1, dynamin-related protein 1; EDH, endothelium-dependent hyperpolarization; eNOS, endothelial nitric oxide synthase; GLP-1, glucagon-like peptide-1; NLRP3, NOD-like receptor family pyrin domain-containing 3; NO, nitric oxide; ROS, reactive oxygen species; SGLT2, sodium–glucose cotransporter 2; VSMC, vascular smooth muscle cell.

## Data Availability

No new data were created or analyzed in this study. Data sharing is not applicable to this article.

## Abbreviations

The following abbreviations are used in this manuscript:

ACE	Angiotensin-converting enzyme
ACh	Acetylcholine
AMPK	AMP-activated protein kinase
ANOCA	Angina with non-obstructive coronary arteries
AR	Androgen receptor
ARB	Angiotensin receptor blocker
BH_4_	Tetrahydrobiopterin
CAD	Coronary artery disease
CD31	Cluster of differentiation 31
CD105	Cluster of differentiation 105
CD62E	Cluster of differentiation 62E
cf-mtDNA	Circulating cell-free mitochondrial DNA
CFR	Coronary flow reserve
cGAS	Cyclic GMP-AMP synthase
cGAS-STING	Cyclic GMP-AMP synthase-stimulator of interferon genes
CKD	Chronic kidney disease
CMR	Cardiac magnetic resonance
CMD	Coronary microvascular dysfunction
DAMP	Damage-associated molecular pattern
DRP1	Dynamin-related protein 1
EDH	Endothelium-dependent hyperpolarization
eNOS	Endothelial nitric oxide synthase
ESC	European Society of Cardiology
GLP-1	Glucagon-like peptide-1
H_2_O_2_	Hydrogen peroxide
HMR	Hyperemic microvascular resistance
ICFT	Invasive coronary function testing
IMR	Index of microvascular resistance
INOCA	Ischemia with non-obstructive coronary arteries
LDL	Low-density lipoprotein
LOX-1	Lectin-like oxidized low-density lipoprotein receptor-1
MFR	Myocardial flow reserve
mtDNA	Mitochondrial DNA
mtROS	Mitochondrial reactive oxygen species
oxLDL	Oxidized low-density lipoprotein
PET	Positron emission tomography
PGC-1α	Peroxisome proliferator-activated receptor gamma coactivator 1-alpha
ROS	Reactive oxygen species
SGLT2	Sodium–glucose cotransporter 2
STING	Stimulator of interferon genes
TLR9	Toll-like receptor 9
TNF-α	Tumor necrosis factor-alpha
VSMC	Vascular smooth muscle cell
